# Comparison of different treatment strategies in the management of endogenic caesarean scar pregnancy: a multicentre retrospective study

**DOI:** 10.1186/s12884-022-04633-y

**Published:** 2022-05-12

**Authors:** Wenjie Qu, Hua Li, Teng Zhang, Yuan Zhang, Yanli Ban, Ningfeng Li, Jingyan Jiang, Juan Xie, Wentian Shi, Yiping Hao, Ruowen Li, Wei Liu, Baoxia Cui

**Affiliations:** 1 Department of Gynaecology and Obstetrics, Qilu Hospital of Shandong University, No.107 Wenhua West Road, Jinan, Jinan, 250012 China; 2 Department of Gynaecology and Obstetrics, Taian Central Hospital, No.29 Longtan Road Taishan District, Tai’an City, Jinan, 250012 China; 3 Cheeloo College of Medicine, Shandong University, No.44 Wenhua West Road, Jinan, Jinan, 250012 China; 4 Department of Obstetrics and Gynaecology, Jinan Central Hospital, No.105 Jiefang Road, Jinan, Jinan, 250012 China; 5 Obstetrics and Gynaecology Department, Liaocheng People’s Hospital, NO.67 Dongchang West Road, Liaocheng City Jinan, 250012 China

**Keywords:** Caesarean scar pregnancy, Endogenic, Methotrexate, Curettage, Hysteroscopy

## Abstract

**Background:**

The aim of this study was to evaluate the effectiveness and safety of different treatment strategies for endogenic caesarean scar pregnancy (CSP) patients.

**Methods:**

According to Vial’s standard, we defined endogenic-type CSP as (1) the gestational sac growing towards the uterine cavity and (2) a greater than 0.3 cm thickness of myometrial tissue at the caesarean scar. A total of 447 endogenic CSP patients out of 527 patients from 4 medical centres in China were enrolled in this study. A total of 120 patients were treated with methotrexate (MTX) followed by surgery, 106 received ultrasound-guided curettage directly and 221 received curettage combined with hysteroscopy. The clinical information and clinical outcomes of these patients were reviewed. Successful treatment was defined as (1) no additional treatment needed, (2) no retained mass of conception and (3) serum β subunit of human chorionic gonadotropin (β-hCG) level returning to a normal level within 4 weeks. The success rate was analysed based on these factors.

**Result:**

Among 447 patients, no significant difference was observed in baseline characteristics between groups except for foetal heartbeat. The success rate was significantly different (*p*<0.001) among the three groups. The highest success rate of 95.9% was noted in the hysteroscopy group, and the lowest success rate of 84.0% was noted in the curettage group. In addition, the MTX group reported the longest hospital stay and highest expenses, but the curettage group showed the shortest and lowest expenses, respectively. Nevertheless, no difference in blood loss was observed between the groups.

**Conclusion:**

The combination of curettage and hysteroscopy represents the most effective strategy. Pretreatment with MTX did not result in better clinical outcomes. Ultrasound-guided curettage directly should not be considered a first-line treatment choice for endogenic CSP patients.

**Supplementary Information:**

The online version contains supplementary material available at 10.1186/s12884-022-04633-y.

## Background

Caesarean scar pregnancy (CSP) is one of the most severe complications of caesarean delivery with a reported incidence of 1 per 2000 pregnancies [1]. It is defined as a gestational sac that implants into the hysterotomy site of a previous caesarean delivery [2]. The prenatal diagnosis of CSP is commonly accomplished by ultrasound [1]. The diagnosis is based on the presence of a gestational sac at the site of the previous uterine incision and an empty uterine cavity and cervix as well as thin myometrium adjacent to the bladder [3].

In 2000, Vial proposed the classification of CSP into 2 types based the position of the gestational sac: endogenic (type 1) and exogenic (type 2) CSP [4]. The first type is due to the implantation of the amniotic sac on a scar with progression of the pregnancy in the cervico-isthmic space and the uterine cavity, whereas the second is a deep implantation in a caesarean scar defect with progression towards rupture and bleeding. In either type, CSP could lead to severe haemorrhage, uterine rupture and hysterectomy [1, 3]; therefore, pregnancy termination should be considered after diagnosis. A multitude of treatment modalities have been proposed for the management of CSP, including expectance, medicine, uterine artery embolization (UAE), surgery and combination [3]. However, the best approach in terms of patient safety and clinical effectiveness has yet to be determined due to their low prevalence.

Medical treatment is one of the most commonly used treatment methods. Among all medicines, methotrexate (MTX) is most often utilized given its history of use in ectopic pregnancy. It is a folic acid antagonist that inhibits the enzyme dihydrofolate reductase, thereby interfering with DNA synthesis in rapidly dividing cells, such as trophoblasts [5]. However, the use of MTX as the first-line approach for CSP is often associated with a low success rate (50–60%) [6–9], and additional interventions, such as surgery, are often required. As the most convenient and inexpensive method of surgery, curettage is always the first choice after MTX for many doctors. Several studies provided data demonstrating that MTX followed by curettage might represent the best treatment for CSP [10, 11]. On the contrary, studies also provided evidence that MTX combined with curettage resulted in more blood loss, a long duration of hospitalization and a normal β-human chorionic gonadotropin concentration [12].

Doctors previously held the notion that direct surgery without medical pretreatment would lead to massive uncontrolled bleeding. Some studies compared several different strategies in CSP treatment and reported that surgery is also a good choice for CSP for patients with a long gestational age, a large gestational sac diameter, high β-hCG levels, or an ample blood supply [13]. Recently, hysteroscopy has been extensively developed as a minimally invasive surgery and has gradually been considered by many doctors [14, 15]. Given that endogenic CSP has a lower risk of bleeding, direct hysteroscopic surgery is becoming a first-line treatment for many gynecologists.

In this study, we aimed to evaluate three different strategies in the management of endogenic CSP, including MTX followed by surgery, ultrasound-guided curettage and curettage plus hysteroscopy without MTX. For the evaluation, we introduced the success rate, blood loss during surgery, hospital time and expenses to reflect the availability and security of treatments.

## Methods

### Patients

This retrospective study analysed 527 endogenic CSP patients treated at Qilu Hospital, Jinan Central Hospital, Taian City Central Hospital and Liaocheng People’s Hospital, Shandong, China from August 2013 to May 2020. Data were collected through medical records and short-term follow-ups. The study was approved by all the hospitals’ ethics committees. Informed consent from the patients was exempt because of the retrospective nature of the study.

The diagnosis of CSP relies on clinical presentation and sonographic signs. A woman with a positive pregnancy test and prior caesarean delivery should undergo an early transvaginal sonographic (TVS) assessment. The ultrasound criteria include the following: (1) an empty uterus and cervical canal, (2) a gestational sac at the caesarean scar site, (3) a vascular area noted at the previous caesarean scar in Doppler scan, and (4) thin or absent myometrial tissue between the bladder and the gestational sac [16]. According to Vial’s standard and our clinical experience, we defined the endogenic type as (1) the gestational sac growing towards the uterine cavity and (2) the thickness of myometrial tissue at the caesarean scar was no less than 0.3 cm given that a thinner myometrium means a greater possibility of deep implantation. Summarizing the above information, gynaecologists made the final diagnosis of endogenic CSP. This study only included patients in the first trimester because those in the second trimester require a more detailed assessment of placental invasion and other complications. Patients who were unsuccessfully treated and then transferred to these hospitals were also removed. We also excluded patients with other complications, including ovarian or fallopian tube cysts and uterine myomas. Finally, a total of 447 endogenic CSP patients were enrolled in this study.

### Treatments

The criteria of selecting treatment strategies for CSP patients without life-threatening situations in these departments plurally combined symptoms, Ultrasonic findings, laboratory indicators and patients’ wishes. Patients could have several choices including expectant management, medical treatment, uterine artery embolization (UAE) and different kinds of surgeries. In this study, we included patients treated with MTX followed by surgery, which was defined as the MTX group, and surgery directly. The surgery included ultrasound-guided curettage only (defined as the curettage group) and curettage combined with hysteroscopy (defined as the hysteroscopy group). MTX was administered intramuscularly at a dose of 50 mg/m^2^ body surface area or interventionally through the uterine artery at a total dose of 50 mg. Then, the surgery was performed after a conspicuous decline in the serum β subunit of human chorionic gonadotropin (β-hCG), which typically occurred within 1 week after MTX treatment. However, surgery was performed as soon as patients were diagnosed in the curettage and hysteroscopy group. Curettage and hysteroscopy were performed by well-trained gynaecological surgeons. Before any surgery, 6 units of pituitrin diluted in 20 mL saline were injected into 3 and 9 points of the cervix. In both curettage group and hysteroscopy group, curettage was performed under ultrasound monitoring with a negative pressure of 400–500 mmHg. After suction, it was necessary to check the villi and embryonic tissue in the container. In the hysteroscopy group, hysteroscopy was performed by experienced gynecologists after curettage to examine whether residual pregnancy tissue was present in the uterine scar site. Hysteroscopy was performed under general anaesthesia with the woman in dorsolithotomy position. The main surgical procedures are similar to that of Wang et al. [17]. Uterine distension was achieved using 5% mannitol solution with a pressure <100 mmHg. The intervention began by an overview of the uterine cavity. The endometrial cavity was empty and a diverticulum could be seen at the scar of the anterior wall of the uterus. The residual pregnancy tissue was excised by wire loop electrode in an 80 W monopole current and blood vessels were coagulated in a 100 W coagulation current.

### Data collection

We defined a successful treatment as (1) no additional treatment needed, (2) no retained mass of conception and (3) serum β-hCG level returning to a normal level within 4 weeks. The success rate is equal to the number of patients who are successfully treated over the total number of patients under a certain treatment. The clinical characteristics of the patients, including age (year), number of previous caesarean sections, years from last caesarean section, gestational age (day), foetal heartbeat activity, vaginal bleeding, abdominal pain, myometrial thickness of uterine scar (mm) and gestational sac mass diameter (cm), were collected. The gestational sac/mass diameter was defined as the largest length of the sac or mass. Serum β-hCG levels (mIU/ml) before and after treatment, time of surgery (min), blood transfusion and blood loss during surgery (ml) were collected to evaluate the safety and availability of surgery. Blood loss during surgery was assessed by the surgeons according to blood volume in the container and sterile pads. Postoperative serum β-hCG value was measured on the second day after surgery. Beyond that, weekly measuring of serum β-hCG levels and TVS were required until serum β-hCG levels returned to normal levels and no mass was found in the uterine cavity. Here, β-hCG differences between treatments, rate of β-hCG decline, time of hospital stay and hospitalization expenses were also collected to evaluate the availability of different treatments. The β-hCG difference is calculated based on the serum β-hCG level before a specific treatment minus that after treatment. The rate of β-hCG decline was equal to the β-hCG difference over the initial value before treatment. Hospitalization expenses include expenditures for medicine, surgery and nurse care during hospital stays.

### Statistical analysis

Statistical analysis was performed using the Statistical Package for the Social Sciences (SPSS, Chicago, USA) version 25.0 software. Categorical variables are presented as numbers and percentages, and between-group differences were assessed by the chi-squared test. Normality tests were performed for all quantitative variables, and none of the variables exhibited a normal distribution. Therefore, all quantitative data are presented as the median (interquartile range) and were assessed by nonparametric tests. A *P* value less than 0.05 was considered indicative of a statistically significant difference.

## Results

### Baseline characteristics among patients in the three groups

A total of 447 endogenic CSP patients were enrolled in this study (see Additional file 1). Among these patients, 120 (26.85%) patients were treated with MTX followed by surgery, 106 (32.42%) were treated with ultrasound-guided curettage, and 221 (67.58%) were treated with curettage combined with hysteroscopy. The baseline characteristics of these patients in the three groups are described in Table 1. Foetal heartbeat significantly differed among the three groups. There were no significant differences with respect to age, number of previous caesarean sections, years from last caesarean section, vaginal bleeding, abdominal pain, gestational age, gestational sac/mass diameter, myometrial thickness of uterine scar, or serum β-hCG and haemoglobin (HGB) levels before surgery.

**Table 1:** Comparison of baseline characteristics among patients in three groups

		MTX group (*n* = 120)	Curettage group (*n* = 106)	Hysteroscopy group (*n* = 221)	*P* value
Age (years)		34.0 (30.0, 38.0)	33.0 (29.0, 36.0)	33.0 (30.0, 37.0)	0.472
Number of caesarean sections					
	1	66(55.0%)	59 (55.7%)	123 (55.7%)	0.999
	2	50(41.7%)	43 (40.6%)	91 (41.2%)	
	3	4(3.3%)	4 (3.8%)	7 (3.2%)	
Years from last caesarean		5.0 (2.0, 9.0)	4.0 (2.0, 7.0)	4.0 (2.1, 7.8)	0.369
Vaginal bleeding	Yes	73 (60.8%)	54 (50.9%)	132 (59.7%)	0.243
	No	47 (39.2%)	52 (49.1%)	89 (40.3%)	
Abdominal pain	Yes	25 (20.8%)	27 (25.5%)	52 (23.5%)	0.706
	No	95 (79.2%)	79 (74.5%)	169 (76.5%)	
Gestational age (days)		49.0 (42.0, 56.0)	49.0 (42.0, 62.0)	49.5 (42.3, 59.0)	0.914
Gestation sac/mass diameter (cm)		2.6 (1.8, 3.5)	2.6 (1.7, 4.1)	2.3 (1.7, 3.2)	0.096
Foetal heartbeat	Present	34 (28.3%)	38 (35.8%)	87 (39.4%)	< 0.001
	Absent	72 (60.0%)	62 (58.5%)	132 (59.7%)	
	Missing	14 (11.7%)	6 (5.7%)	2 (0.9%)	
Myometrial thickness of uterine scar (mm)		3.7 (3.0, 4.2)	3.8 (3.0, 5.0)	3.8 (3.0, 4.6)	0.153
β-hCG before treatment (mIU/ml)		19,486.0 (3039.6, 51,123.3)	22,543.0 (12,817.0, 60,586.3)	24,493.5 (10,371.8, 46,995.0)	0.184
HGB before surgery (g/L)		125.0 (114.5, 135.0)	124.0 (119.0, 133.0)	122.0 (113.0, 130.0)	0.102

There were no significant differences with respect to baseline characteristics except foetal heartbeat

### Clinical outcomes for the three groups

Characteristics reflecting the availability and security of three different strategies are compared in Table 2. According to our definition, the success rates of the three groups are 85.8, 84.0 and 95.9%. In addition, success treatment, surgery time, hospital time, hospitalization expenses, β-hCG after surgery, and rate of β-hCG decline (*P* < 0.05) were significantly different among these groups. No significant differences were observed with regard to blood loss during surgery, blood transfusion or β-hCG difference between treatments.

**Table 2:** Comparison of outcomes among the three groups

	MTX group (*n* = 120)	Curettage group (*n* = 106)	Hysteroscopy group (*n* = 221)	*P* value
Treatment success	103 (85.8%)	89 (84.0%)	212 (95.9%)	< 0.001
Surgery time (min)	20.0 (10.0 30.0)	20.0 (15.0 30.0)	25.0 (20.0 30.0)	0.001
Blood loss (ml)	20.0(10.0, 50.0)	20.0 (10.0 20.0)	20.0 (10.0 30.0)	0.455
Blood transfusion Yes	1 (0.8%)	3 (2.8%)	4 (1.8%)	0.513
No	119 (99.2%)	103 (97.2%)	217 (98.2%)	
β-hCG after treatment (mIU/ml)	1462.5 (307.443757.57)	4611.00 (1125.009713.00)	5108.00 (2213.509978.50)	< 0.001
β-hCG difference (mIU/ml)	17,562.10 (2945.7046044.62)	19,640.00 (9506.0054520.85)	17,305.00 (5929.1534914.25)	0.629
Rate of β-hCG decline (%)	91.73 (84.45 96.38)	86.46 (74.26 92.43)	78.07 (68.37 85.14)	< 0.001
Hospital time (days)	9.0 (7.0, 16.0)	3.0 (2.0 5.0)	4.0 (3.0 5.0)	< 0.001
Hospitalization expenses (¥)	13,677.50 (8240.75, 16,760.75)	4005.70 (3390.075632.22)	11,640.68 (10,746.3312684.02)	< 0.001

Success treatment, surgery time, hospital time, hospitalization expenses, β-hCG after surgery, and rate of β-hCG decline (*P* < 0.05) were significantly different among these groups

### Complications

Among the 17 patients in the MTX group who were not successfully treated, one patient underwent a curettage operation, and the remaining patients received medical treatment, including repeated MTX injection and the administration of a contraction-promoting drug (such as misoprostol), until serum β-hCG levels returned to normal. All 17 patients in the curettage group who required greater than 4 weeks to return to normal β-hCG levels received mifepristone or a contraction-promoting drug. Among the 9 patients in the hysteroscopy group who were defined as unsuccessfully treated, one patient underwent a repeated hysteroscopy operation, and the remainder received medicine. No cases of uterine rupture, bladder injury, hysterectomy and haemorrhagic shock were noted in these patients.

## Discussion

CSP was first reported by Larsen and Solomon and refers to a special type of ectopic pregnancy in which embryos are implanted on the uterine scar [18]. Given the significant increase in the percentage of caesarean deliveries, CSP diagnosis has increased and represents a challenge for contemporary obstetrics [19]. The high risk of death and serious complications leads to heated discussion about the management strategies for CSP. However, no internationally recognized diagnosis and treatment program has been published thus far.

In this study, the clinical information of 447 patients was collected, and a significant difference was observed between the three groups. Among the three different management options, the highest success rate of 95.9% was found in the curettage plus hysteroscopy group followed by 85.8% in the MTX group and 84.0% in the ultrasound guided curettage group. These results were similar to previous studies [13, 20]. However, previous studies were limited by their small number of cases. The surgery time in the hysteroscopy group was relatively longer. A more careful operation may be considered in the surgery group, and easer surgical options were chosen after MTX pretreatment given that doctors used to believe that MTX pretreatment would reduce the amount of bleeding. However, the likelihood of MTX completely inactivating trophoblast cells is very low. Under this premise, the choice of a non-visible or uncertain surgery method after MTX treatment was not optimal. This traditional concept may be responsible for the lowest success rate in the MTX group. According to our results, the β-hCG level between different treatments showed no difference, but the rate of β-hCG decline in the MTX group was significantly greater than that in both surgery groups. This result indicates that the β-hCG value in the MTX group declined more despite the much longer hospital stay. Due to the time needed by doctors to observe the patient between MTX treatment and surgery, the MTX group showed significantly the longest hospital time and the highest expenses. Similarly, previous study even reported the long duration of hospitalization of 19.38 ± 8.75 days in MTX pretreatment group [12]. This result demonstrates that that MTX treatment was not highly efficient, although a greater reduction in β-hCG levels was observed. The curettage group demonstrated the shortest hospital stay, lowest expenses and a relatively greater reduction in β-hCG levels. However, this is not an optimal strategy given that it exhibited the lowest success rate. Interestingly, the surgery group, especially the hysteroscopy group, reported a significantly higher rate of foetal heartbeat but no difference in blood loss, indicating that foetal heartbeat presence may unwittingly affect the choice of different treatment options but actually does not influence the amount of blood loss or the safety of treatments. Considering that no study has supported foetal heartbeat as an independent risk factor so far, more clinical research was urgently need to explain the connection between foetal heartbeat and treatment options.

To the best of our knowledge, this is the first study concentrating on endogenic CSP patients and providing detailed success rates for three different strategies. According to our results, patients pretreated with MTX showed a lower success rate, longer hospital stay, and higher expenses but proportionable blood loss compared with those in the hysteroscopy group. This result demonstrates that MTX pretreatment did not result in better outcomes. Moreover, endogenic CSP patients treated with ultrasound-guided curettage only showed the lowest success rate of 84.0%, which was similar to previous studies in CSP patients [20–22]. Therefore, we concluded that curettage combined with hysteroscopy tends to be the most effective strategy, but curettage alone should not be considered a first-line treatment choice for endogenic CSP patients. In addition, past work on CSP was invariably limited by the small sample size. The limited number of cases is associated with a greater possibility of selection bias and a worse generalization ability of the results. In this study, a large number of patients treated at four medical centres were analysed, which strengthened our conclusion from a statistical point of view.

There were also several limitations in this study. First, given its retrospective nature, detailed information was occasionally not available. For example, the blood loss in our study was assessed mainly by surgeons, so it was simply not evaluated as accurately as expected, which might lead to our result indicating no significant difference between groups. In addition, due to the absence of a standard surgical procedure for CSP, it is possible that the skill and experience of different surgeons affect our conclusion. These findings also support the urgent need for an optimal treatment consensus and gynaecologists specializing in CSP.

Above all, our study provided an accurate success rate of different treatment strategies for endogenic CSP patients by analysing the clinical outcomes of 447 cases. Our results demonstrated that curettage followed by hysteroscopy could be a recommended safe strategy with possible advantages. However, further studies including a large series with comprehensive information, detailed follow-up data and multiple patient sources are needed to support this finding. Medical departments specializing in CSP have been established at our institutions, and detailed treatment as well as integrated follow-up data of CSP patients are being collected. Thus, more convincing studies and prospective research will be provided in the future.

## Conclusions

Caesarean scar pregnancy is one of the most severe complications of caesarean delivery with no internationally recognized treatment program. Endogenic Caesarean scar pregnancy could also lead to severe haemorrhage, uterine rupture and hysterectomy. Thus, the security and availability of surgery should be fully considered for endogenic caesarean scar pregnancy patients. Analysing clinical data from 447 endogenic CSP patient, we came to the conclusion that the combination of curettage and hysteroscopy represents the most effective strategy, pretreatment with MTX did not result in better clinical outcomes.

## Supplementary Information

**Additional file 1.**

## Data Availability

All data generated or analysed during this study are included in this published article and its supplementary information files.

## Abbreviations

CSP: Caesarean scar pregnancy
MTX: Methotrexate
UAE: Uterine artery embolization
TVS: Transvaginal sonographic
β-hCG: β subunit of human chorionic gonadotropin
SPSS: Statistical Package for the Social Sciences
HGB: haemoglobin
